# Broad Learning Enhanced ^1^H-MRS for Early Diagnosis of Neuropsychiatric Systemic Lupus Erythematosus

**DOI:** 10.1155/2020/8874521

**Published:** 2020-11-22

**Authors:** Yan Li, Zuhao Ge, Zhiyan Zhang, Zhiwei Shen, Yukai Wang, Teng Zhou, Renhua Wu

**Affiliations:** ^1^Department of Medical Imaging, The 2nd Affiliated Hospital, Shantou University Medical College, Shantou 515041, China; ^2^Department of Computer Science, Shantou University, Shantou 515041, China; ^3^Department of Medical Imaging, Huizhou Central Hospital, Huizhou 516000, China; ^4^Department of Rheumatology and Immunology, Shantou Central Hospital, Shantou 515041, China; ^5^Key Laboratory of Intelligent Manufacturing Technology (Shantou University), Ministry of Education, Shantou 515063, China

## Abstract

In this paper, we explore the potential of using the multivoxel proton magnetic resonance spectroscopy (^1^H-MRS) to diagnose neuropsychiatric systemic lupus erythematosus (NPSLE) with the assistance of a support vector machine broad learning system (BL-SVM). We retrospectively analysed 23 confirmed patients and 16 healthy controls, who underwent a 3.0 T magnetic resonance imaging (MRI) sequence with multivoxel ^1^H-MRS in our hospitals. One hundred and seventeen metabolic features were extracted from the multivoxel ^1^H-MRS image. Thirty-three metabolic features selected by the Mann-Whitney *U* test were considered to have a statistically significant difference (*p* < 0.05). However, the best accuracy achieved by conventional statistical methods using these 33 metabolic features was only 77%. We turned to develop a support vector machine broad learning system (BL-SVM) to quantitatively analyse the metabolic features from ^1^H-MRS. Although not all the individual features manifested statistics significantly, the BL-SVM could still learn to distinguish the NPSLE from the healthy controls. The area under the receiver operating characteristic curve (AUC), the sensitivity, and the specificity of our BL-SVM in predicting NPSLE were 95%, 95.8%, and 93%, respectively, by 3-fold cross-validation. We consequently conclude that the proposed system effectively and efficiently working on limited and noisy samples may brighten a noinvasive *in vivo* instrument for early diagnosis of NPSLE.

## 1. Introduction

Systemic lupus erythematosus (SLE) is an autoimmune disease involving multiple organs or systems, such as the central nervous system (CNS), peripheral nervous system (PNS), skin, joints, and kidneys [1]. Up to 75% of SLE patients suffer from CNS and PNS disorder [2]. The neuropsychiatric systemic lupus erythematosus (NPSLE) is closely related to a worse prognosis and a serious mortality [3–5]. In 1999, a classification criterion for NPSLE has been developed by the American College of Rheumatology (ACR), which included case definitions for 19 neuropsychiatric syndromes, significant exclusions, and recommendation of ascertainment [6]. It is still a tough task to ascribe a specific symptom or sign to NPSLE.

Magnetic resonance imaging (MRI) is widely considered a promising noninvasive tool for SLE diagnosis [7]. Conventional MRI sequences, including T1-weighted, T2-weighted, T2 fluid-attenuated inversion recovery (T2-FLAIR) images, and diffusion-weighted imaging (DWI) can sensitively reveal abnormal changes caused by axonal damage, cortical damage, cerebral atrophy, cerebral infarctions, inflammatory-like lesions, and small vessel disease [8–10]. However, there were about 50% of NPSLE patients that had normal intensities in the structural MRI [11], displaying the limitation of conventional MRI.

Proton magnetic resonance spectroscopy (^1^H-MRS) is accessible to the levels of tissue metabolites including N-acetylaspartate (NAA), creatine (Cr), choline (Cho), glutamate (Glu), Glu-glutamine (Gln), myo-inositol (mI), and lactate (Lac) [12–14].

N-Acetylaspartate (NAA) is a special character of neural cells, and a decreased peak of NAA in MRS spectra represents the reduction of neurons [15–17]. Choline (Cho) plays an important role in generating the phospholipid of the cell membrane. An elevated Cho peak shows the increased cell membrane synthesis, which reflects that the cell structure is in a mess [16]. Glutamate (Glu) and Glu-glutamine (Gln) are related to glutamatergic neurotransmitters, and increased Glu and Gln represent a high risk in psychosis [17]. Myo-inositol (mI) is involved in glial metabolism, and increased mI reflects glial involvement [18]. Moreover, ^1^H-MRS enables quantifying metabolic concentration noninvasively *in vivo* [19]. Thus, it has been widely explored in pioneering researches of noninvasively diagnosing NPSLE [9, 20, 21]. In previous studies, a reduction in NAA was observed, whereas total Cho (tCho) and mI are raised, in normal appearing brain tissue of NPSLE patients [20], which demonstrated that neural biomarkers were able to predict the early involvement of the central nervous system in SLE. However, Zimny et al. found that the levels of mI and Cho were almost normal in their patients with SLE or NPSLE [22].

Therefore, these changes detected by single-voxel MRS in the above studies may not be specific enough, since there is limitation of one voxel which is not included in all the regions that may have pathological changes. In this regard, the conventional statistical methods easily fail to distinguish NPSLE due to the individual difference of metabolic features among limited samples. The accuracy of diagnosing NPSLE by ^1^H-MRS required further improvement by emerging machine learning techniques.

Deep neural networks have been successfully applied in a great number of applications [23], including medical image processing [24–26]. However, there are some unavoidable systematic errors, such as relaxation and partial-volume effects, which resulted in missing metabolic values. Consequently, a sufficient and consistent training set can hardly be constructed, which makes a great challenge to apply deep techniques to this task, since the samples are too small to train a deep structure with a great number of parameters. Moreover, the metabolic features mingling with missing values and noise also burden the classifier to make a correct judgement.

To develop a robust and effective model to quantitatively analyse the metabolic features or the nonlinear combination of metabolic features of the NPSLE patients, we rethink the potential of a support vector machine (SVM), which has been regarded as a succinct model to separate complicated data in limited samples and able to optimize convexly [27]. We also draw the idea of the broad learning system [28] to construct a shallow but effective learning system to extract discriminable features by shallow structures in a layer-wise mechanism. The proposed support vector machine broad learning system (BL-SVM) was applied to a retrospective analysis of the metabolic features screened by ^1^H-MRS quantitatively. Although the samples in the training set were limited, the SVMs embedded in the BL-SVM can still learn from the metabolic features layer-wise optimally. The diversity of each SVM is increased by resampling the training using the bootstrap method to enhance the robustness of the learning system. The results have confirmed that the metabolic features screened by multivoxel magnetic resonance spectroscopy can be used to quantitatively distinguish NPSLE patients from the healthy controls. Our findings may brighten an automatic and noninvasive computer-aided diagnostic instrument for NPSLE at an early stage.

## 2. Materials and Methods

### 2.1. Patients and Controls

This retrospective study has been approved by the Research Ethics Committee of the 2nd Affiliated Hospital, Shantou University Medical College. Informed consent was obtained from all subjects previously. The identifiers of the subjects were removed before analysis. The ^1^H-MRS data from 23 NPSLE patients and 16 age-matched healthy controls (HCs) were obtained at the Department of Rheumatology and Immunology of Shantou Central Hospital and the Department of Endocrinology and the Medical Examination Center of the 2nd Affiliated Hospital, Medical College of Shantou University, during April 2014 to March 2015. The inclusion criteria were as follows: (1) The group of NPSLE patients was diagnosed according to the revised 1997 American College of Rheumatology (ACR) criteria and the 1999 ACR definitions for NPSLE. All clinical manifestations were obtained at the baseline visit by a careful medical record review. In this group, patients had at least one neuropsychiatric complaints. (2) HCs did not have any neurologic, psychiatric, or systemic diseases, which would influence the results of multivoxel ^1^H-MRS, and none of them uses any psychoactive medication. (3) All the subjects underwent both conventional MRI examination and multivoxel ^1^H-MRS examination in our hospitals. (4) The clinical characteristics of all patients were available.

### 2.2. Magnetic Resonance Imaging

All subjects underwent MR imaging using a 3.0 T system (SIGNA, General Electric Medical Systems) with an eight-channel standard head coil. The repetition time (TR) of T2-weighted imaging was 4420 ms. The echo time (TE) was 112.1 ms. The slice thickness was 5 mm with a 1 mm gap. The matrix size was 512 × 512. The field of view (FOV) was 160 × 160 mm^2^. The parameters of T2-weighted imaging are listed in Table 1.

The multivoxel ^1^H-MRS was based on a point-resolved spectroscopy sequence (PRESS) with a two-dimensional multivoxel technique. The TR of the multivoxel ^1^H-MRS was 1500 ms. The TE was 35 ms. The number of excitations (NEX) was 1. The phase × frequency = 18 × 18, and the volumesofinterest(VOIs) = 10 × 8 × 2cm^3^. The VOIs of ^1^H-MRS were placed on the T2-weighted images including the entire basal ganglion level. The parameters of multivoxel ^1^H-MRS are listed in Table 2.

### 2.3. Imaging Processing

The acquired spectroscopy data were firstly preprocessed by a SAGE software package (GE Healthcare) to correct the phase and frequency. Then, commercially available automatic LCModel software (LCModel Inc., Canada, version 6.2-2B) was used to fit the spectra, correct the baseline, relaxation, and partial-volume effects, and quantify the concentration of metabolites. Furthermore, we used the absolute NAA concentration in single-voxel MRS as the standard to gain the absolute concentration of metabolites. After that, the NAA concentration of the corresponding voxel of multivoxel MRS was collected consistently. The spectra would be accepted if the signal-to-noise ratio (SNR) is greater than or equal to 10 and the metabolite concentration with standard deviations (SD) is less than or equal to 20%. Notice that every individual metabolite has its basis spectra, even if the metabolites are hardly separated, such as NAA and N-acetylaspartylglutamate (NAAG). However, the linear combination of similar spectra of metabolic concentrations is more accurate than the individual concentrations. In this regard, we list the linear combination, together with their %SD values, in the concentration table. Concentration ratios are not easily affected by water scaling and less sensitive to relaxation and partial-volume effects. Thus, we extracted the absolute metabolic concentrations, the corresponding ratio, and the linear combination of the spectra from different brain regions, which were RPCG, LRCG, RDT, RDT, LDT, RLN LLN, RPWM, and LPWM.

### 2.4. Quantitative Analysis via a SVM-Based Broad Learning System

The computer-aided analysis not only is user-friendly, rapid, and low-cost for learning and operation but also avoids clinical subjective judgment [25]. Building deeper neural networks has attracted increasing interests from academies and industries [23]. However, the neural network-based deep models still suffer from nonconvex optimization, unfriendly paralleling, and uninterpretable issues [27]. In particular, when the training samples are limited, the neural network-based deep models tend to overfit the training set; e.g., the model remembers what all training samples are exactly alike but fails to distinguish the samples which they have never seen. In this regard, the conventional deep neural networks are not compatible with this task. Thus, we formulate this retrospective analysis as a classification problem, so we develop a support vector machine broad learning system (BL-SVM) for quantitative analysis to distinguish the NPSLE patients from control ones. Unlike backpropagate deep stacked architecture, the BL-SVM organizes support vector machines (SVMs) in a shallow but broad scheme. The SVMs in each layer optimally extract the data representation layer-wise even if the training samples are limited, which ensures the antisaturation property. Different from a BP-like tuning scheme developed by Wang et al. [27], our BL-SVM enables fast learning for each SVM in each layer simultaneously and without time consumption for backpropagating iteratively.

The features involved in this model were selected by recursive feature elimination (see [29] for more details). Then, we construct a training set *T* = {(*x*_*k*_, *y*_*k*_}, where *x* ∈ *ℝ*^*n*^, *y* ∈ {−1, +1}, *k* = 1 ⋯ *K*, and *K* is the total number of training samples. We denote the *i*th SVM in the *l*th layer as svm(*l*, *i*), which can be trained by optimizing

(1) $$\begin{array}{c} \begin{array}{cc} & \underset{\omega ,b}{\max}\frac{2}{||\omega ||},\\ \text{s}.\text{t}. & y_{k}\left(\omega^{Τ}x_{k}+b\right)\geq 1,k=1,\cdots ,K. \end{array} \end{array}$$

For each SVM, the further away from a sample from its corresponding hyperplane, the more confident the SVM makes the classification decision. Different from Wang et al. [27], we design a new confidence function, e.g., equation (2), for the *i*th SVM in the *l*th layer, since tanh(·) is continuously differentiable everywhere.

(2) $$\begin{array}{c} y^{\left(l,i\right)}=\tanh\left(\omega^{⊤}x_{k}+b\right). \end{array}$$

The input of the SVMs in *l* + 1th layer is the initial input concatenating the confidence values of all SVMs in previous layers.

(3) $$\begin{array}{c} x^{\left(l+1\right)}={\left\{x^{\left(1,1\right)},\cdots ,x^{\left(1,i\right)},y^{\left(1,1\right)},\cdots ,y^{\left(1,i\right)},y^{\left(2,1\right)},\cdots ,y^{\left(2,i\right)},\cdots ,y^{\left(l,1\right)},\cdots ,y^{\left(l,i\right)}\right\}}^{⊤} \end{array}$$

The *i*th SVM in the *l* + 1th layer takes *x*^(*l* + 1)^ as input to calculate the confidence value *y*^(*l* + 1, *i*)^. Then, we train the SVMs layer-wise. In each layer, we resample the training set for each SVM to increase the diversity of the individual SVM using the bootstrap method proposed by Zhou et al. [30].

In this study, the number of layers is set to 5. We use 3-fold cross-validation, which is a common resampling procedure in machine learning, to evaluate the performance of our metabolism-based diagnosis model, since it generally results in a less biased or less optimistic estimate of the model skill than other methods, such as a simple train/test split, especially on a dataset with limited samples. Twenty-three NPSLE patients and sixteen healthy controls were randomly divided into 3-fold. The cross-validation was conducted 50 times. For each run, 2 out of 3 subjects were selected for training, and the rest was used for testing, using different random seeds. We calculated the accuracy, sensitivity, and specificity to evaluate the performance of our model.

## 3. Results

### 3.1. Demographics

Twenty-three NPSLE patients and sixteen healthy controls met the inclusion criteria and satisfied the spectra quality detailed in our previous study [9].  Table 3 summarizes the number of NPSLE patients presenting with neuropsychiatric manifestations, including myelitis, seizure disorder, severe headache, stroke, peripheral polyneuropathy, acute confusional state, and anxiety in this study.

There was no significant difference in age between NPSLE patients and the HC set (*p* = 0.765). Obviously, SLE was closely related to gender (*p* = 0.018), and 79% of the patients were females in our study. Although there was a significant difference between NPSLE and HCs, the performance of predicting NPSLE would not be influenced. As we used 3-fold cross-validation to evaluate the accuracy of our BL-SVM system, every subject had the chance to be one of the training set. Results for their demographic characters are summarized in Table 4.

### 3.2. Metabolic Features from Multivoxel ^1^H-MRS

**Figure 1:**
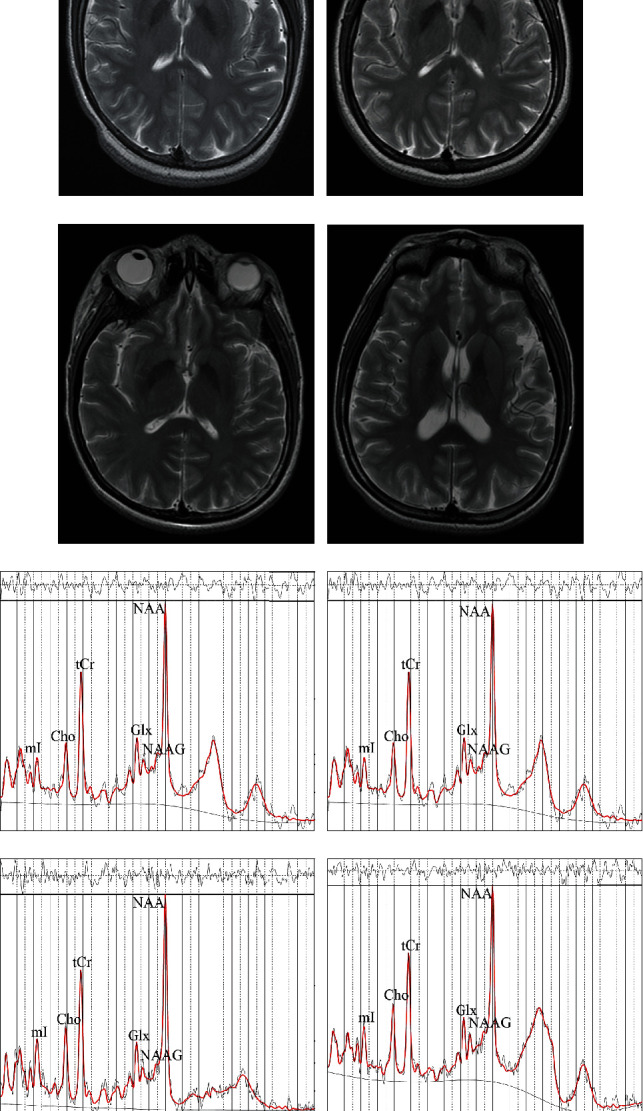
(a) and (b) are the axial view of the basal ganglion slice obtained by multivoxel MRS from HCs, whereas (c) and (d) are from NPSLE patients. (e) and (f) are the attained spectra of the volumes by LCModel from HCs corresponding to the axial view (a) and (b), respectively, whereas (g) and (h) are from NPSLE patients corresponding to the axial view (c) and (d), respectively. NAA is the abbreviation for N-acetylaspartate, NAAG is the abbreviation for N-acetylaspartylglutamate, Cho is the abbreviation for choline, tCr is the abbreviation for total creatine, mI is the abbreviation for myo-inositol, and Glx is the abbreviation for glutamine+glutamate. The chemical shift values in (e)–(h) ranging from 4.0 ppm (leftmost) to 0.2 ppm (rightmost) are divided into 38 equally spaced intervals, e.g., 1 ppm per interval. From (a) to (h), the visual differences of the axial view of the basal ganglion slices and the spectra of the volumes from NPSLE patients and HCs are subtle.

We collected metabolic data from the bilateral posterior cingulate gyrus (PCG), dorsal thalamus (DT), lentiform nucleus (LN), and posterior paratrigonal white matter (PWM), as well as from the right insula (RI) in all subjects. The metabolic features include creatine (Cr), phosphocreatine (PCr), Cr+PCr, NAA, N-acetylaspartylglutamate (NAAG), NAA+NAAG, NAA+NAAG/Cr+PCr ratio, myo-inositol (mI), mI/Cr+PCr, glycerophosphocholine (GPC/Cho)+phosphocholine (Pch), Cho+Pch/Cr+PCr, glutamate (Glu)+glutamine (Gln), and Glu+Gln/Cr+PCr. All brain regions and metabolic features were combined into 117 metabolic features as shown in Table 5. Thirty-three features were found with significant difference (*p* < 0.05) between NPSLE patients and HCs: PCr and Cho+PCh in the right PCG; NAA+NAAG, NAA+NAAG/Cr+PCr, and mI in the left PCG; PCr, Cr+PCr, NAA, NAA+NAAG/Cr+PCr, mI/Cr+PCr, and Cho+PCh in the right DT; NAAG, NAA+NAAG/Cr+PCr, mI, mI/Cr+PCr, Cho+PCh, and Glu+Gln in the left DT; Cr, PCr, mI, Cho+PCh, and Cho+PCh/Cr+PCr in the right LN; PCr, mI/Cr+PCr, Cho+PCh, and Cho+PCh/Cr+PCr in the left LN; Cr and NAA in RI; PCr and Cho+PCh/Cr+PCr in the right PWM; and Cr, NAAG, and mI in the left PWM. The corresponding AUC values using these features for quantitative analysis are listed in Table 5. The AUC values generated by mI/Cr+PCr in LDT and the mI/Cr+PCr in RDT are 0.77 and 0.76, respectively, which achieve the best performance for diagnosing NPSLE among the evaluated features. Obviously, as shown in Figure 1, it is hard to distinguish the NPSLE patients and the HCs, whether by structure images or MRS alone.

### 3.3. Metabolic Features for the BL-SVM System

**Figure 2:**
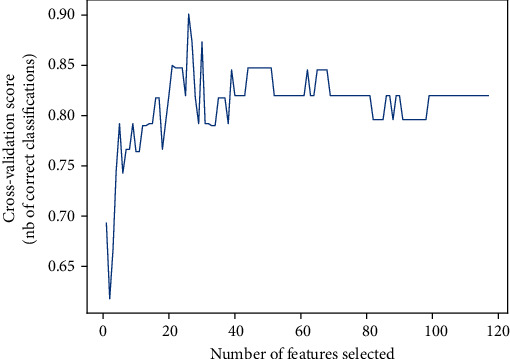
Cross-validation score by the number of features selected by recursive feature elimination.

We employed a feature selection method, e.g., recursive feature elimination [29], to analyse which metabolite or combination of metabolites was closely related to NPSLE and filter out weak features to avoid overfitting. We first built the model on the entire set of metabolite features and computed an importance score for each feature. Then, the least important feature was removed from the current feature set. We retrained the model and computed the important score again. We repeat this step on the feature set until the specified number of features were selected. In the end, we found 26 features that were of the highest importance, as shown in Figure 2. The 26 features were as follows: NAAG, mI/Cr+PCr, and Glu+Gln/Cr+PCr in the right PCG; Cr+PCr, NAA+NAAG, NAA+NAAG/Cr+PCr, mI/Cr+PCr, and Glu+Gln in the left PCG; NAA, NAAG, and Cho+PCh in the left DT; PCr, Cr+PCr, Cho+PCh, Cho+PCh/Cr+PCr, and Glu+Gln/Cr+PCr in the right LN; mI/Cr+PCr, Cho+PCh, and Cho+PCh/Cr+PCr in the left LN; NAA+NAAG/Cr+PCr and Cho+PCh in RI; Cho+PCh/Cr+PCr and Glu+Gln/Cr+PCr in the right PWM; and PCr, NAAG, and NAA+NAAG/Cr+PCr in the left PWM.

**Figure 3:**
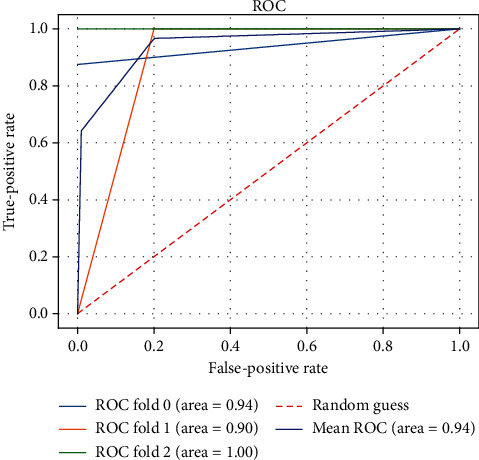
The receiver operating characteristic of the BL-SVM system for diagnosing NPSLE.

However, these features had a complex nonlinear relationship, which made our diagnosis quite challenging. This motivated us to leverage the kernel tricks of the SVM classifier to map the features into a higher dimensional space to make the samples linearly separable. With the selected features, we evaluated the performance of our BL-SVM system on a ROC curve as shown in Figure 3. The AUC, sensitivity, and specificity were 95%, 95.8%, and 93%, respectively.

**Figure 4:**
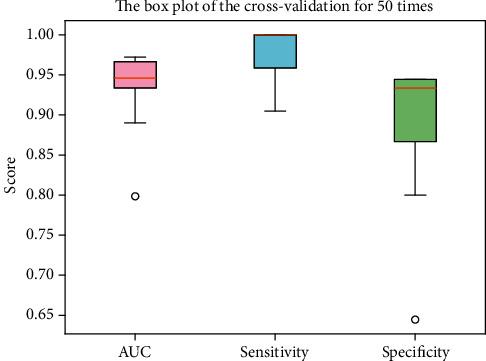
The score of the AUC, sensitivity, and specificity referring to the cross-validation for 50 times.

To estimate the generalization capacity of the proposed model, we perform the cross-validation for 50 times. In each run, we feed a different random seed for the resampling procedure. Two-thirds of samples are for training, and the rest is for testing. The scores of the AUC, sensitivity, and specificity are plotted in a box diagram in Figure 4. The box plot demonstrates that the model is capable of unseeing samples in each run.

## 4. Discussions

In this study, we retrospectively analysed the diagnosis of the NPSLE using multivoxel ^1^H-MRS. Each metabolic feature hardly identified the NPSLE patients from the HCs precisely and was even insignificant to NPSLE. We introduced a support vector machine-based deep-stacked network to quantitatively analyse the metabolic features. The result has shown that this model has good robustness, even there were several missing values of metabolic features caused by the noise of spectra. Furthermore, this model can accurately distinguish NPSLE patients from the HCs, although individual feature does not even manifest statistics significantly, which is better than any single metabolic feature. The results indicated that this model can be a helpful noninvasive computer-aided diagnostic tool for quantitative analysis of NPSLE.

The clinical complications of NPSLE severely affect the patients in their quality of work and life, which also consume a large amount of money. Therefore, the examination methods of early diagnosis and prediction of NPSLE have aroused wide attention; increasingly, laboratory biomarkers and neuroimaging tools have been proposed [31–34].

The production of autoantibodies was used to be a diagnostic biomarker of SLE, and 116 autoantibodies had been found in a literature review using the keywords *autoantibodies* and *systemic lupus erythematosus* [35]. Various autoantibodies are reported that can be used as diagnostic biomarkers, since one of these autoantibodies had a significant difference between NPSLE and SLE patients and healthy controls. Most studies were explorative studies, which were short of repeatability. Segovia-Miranda et al. [36] have confirmed that the antiribosomal P (RP) antibody is related to cognitive dysfunction and other diffuse neuropsychiatric manifestations of NPSLE by altering glutamatergic synaptic transmission in the hippocampus. However, there was a recent study conflicted with their results, which suggested that the anti-P ribosomal antibodies have limited diagnostic value for NPSLE [37].

In this case, advanced neuroimaging technologies were needed urgently. *In vivo* multivoxel MRS allows simultaneously measuring the level of metabolites in several brain regions within a single slice [8] However, a standard for the choice of metabolites and brain regions is not available until now. Single metabolite or the ratio between two metabolites have been used to diagnose NPSLE in most studies [22, 38–40]. There will be numerous exploratory biomarkers for diagnosing NPSLE by applying the previous laboratory and neuroimaging methods. However, accuracy needs to be further improved. Thus, more advanced machine learning methodologies are urgently required.

Broad learning systems are an alternative way to address the time-consuming training process and nonconvex issues, especially when the structure is insufficient to model the system [28]. Support vector machines are a succinct model with convexity optimization property to learn sample-limited data with complicated features [27]. We rethink the potential of SVM in diagnosing NPSLE, and we reconstruct the broad learning system to increase the diversity of features that the SVMs learned. To demonstrate the advantage of SVM-based broad learning systems for diagnosing NPSLE, we compared the BL-SVM with traditional statistical methods that were frequently used for diagnosing NPSLE. Guillen-Del Castillo et al. [41] suggested that the increased mI in normal parietal white matter and parietal white matter demonstrates a strong relationship to the deteriorated prognosis in NPSLE. In our study, the best accuracy of mI in parietal white matter was only 51.6%. For other single metabolites described in previous studies, such as NAA [42], Cho, and Cr [43], used in our model, the best accuracy was 75% in the right DT, 72% in the left PWM, and 77% in the right DT, respectively. It is also not known whether metabolite ratios could improve diagnostic accuracy. Cagonoli et al. proposed that NAA/Cr ratios and Glu/Cr ratios in RI might be biomarkers for NPSLE patients [44]. However, in our study, the accuracy of NAA/Cr ratios and Glu/Cr ratios in RI was both only 50%. Overall, thirty-three features were selected using conventional statistical methods, and the best accuracy among them was only 77%, whereas the BL-SVM system with the metabolic features from multivoxel ^1^H-MRS achieved 95% AUC, 95.8% sensitivity, and 93% specificity, respectively. To confirm that no overfitting occurred in our experiment, we performed 3-fold cross-validation to demonstrate the generalization ability of our BL-SVM system. It is worth noting that there were 26 features of eight brain regions of NPSLE patients that showed the optimal performance to diagnose NPSLE. So we should realize that single ^1^H-MRS may not suitably be used to diagnose NPSLE, because it was restricted to one small region, which missed important pieces of information. What is more, our study confirmed that not only should the absolute concentration of metabolites be considered but also the combination between them, such as NAA+NAAG, Glu+Gln/Cr+Pcr, and mI/Cr+Pcr. The BL-SVM system with the metabolic features from multivoxel ^1^H-MRS as a novel tool should be popularized in the diagnosis of NPSLE, though some studies have combined machine learning and ^1^H-MRS to increase the sensitivity and specificity for distinguishing diseases [14, 45, 46]. However, to the best of our knowledge, the current study is the first to use machine learning-based metabolic features to improve the accuracy of ^1^H-MRS to diagnose NPSLE. Our BL-SVM has achieved a specific performance for the following reasons. First, the kernels map the features into a higher dimensional space to empower the SVMs to split the samples linearly [47], which enables the BL-SVM system to distinguish NPSLE patients from HCs. Secondly, the BL-SVM system can learn to deal with the unavoidable absent metabolic feature value caused by patients moving, partial-volume effect, and overlapping among metabolites, which demonstrated the good robustness of our model. The BL-SVM learning system is general and can be extended to other applications, such as intelligent transportation systems [48–55], intelligent computing [56, 57], and emotion computing [58–60].

### 4.1. Limitations

However, there were some limitations in our study. First, the samples are limited. Although the number of samples is important for evaluating the generalization ability of a model, cross-validation is one of the alternative techniques by resampling to evaluate the generalization capacity of a machine learning model, when we have limited samples. We apply 3-fold cross-validation to evaluate the performance of the presented model 50 times. The results indicate the proposed model capable of unseeing samples. Besides, we have been collecting new samples to evaluate our model and develop new models. Secondly, other advanced medical imaging technologies should be considered to combine with ^1^H-MRS in this system, such as voxel-based morphometry, diffusional kurtosis imaging, and chemical exchange saturation transfer [61–65].

## 5. Conclusion

In this retrospective study, we confirm that the metabolic features obtained by multivoxel proton magnetic resonance spectroscopy can be used to diagnose neuropsychiatric systemic lupus erythematosus by a well-trained support vector machine broad learning system. The support vector machine broad learning system achieves satisfactory AUC, sensitivity, and specificity as 95%, 95.8%, and 93%, respectively. We have also found that the support vector machine broad learning system can even leverage the metabolic features that were not regarded as statistically significant to distinguish the NPSLE patients from HC ones. Furthermore, our support vector machine broad learning system overcame the situation of limited samples with missing metabolic feature values.

In conclusion, the multivoxel proton magnetic resonance spectroscopy enhanced by our support vector machine broad learning system may brighten the computer-aided noninvasive diagnostic instrument for neuropsychiatric systemic lupus erythematosus *in vivo*.

## Figures and Tables

**Table 1:** The parameters of T2-weighted imaging.

Parameters	Value
Repetition time (TR)	4420 ms
Echo time (TE)	112.1 ms
Slice thickness	5 mm
Slice gap	1 mm
Matrix size	512 × 512
Field of view (FOV)	160 × 160 mm^2^

**Table 2:** The parameters of multivoxel ^1^H-MRS.

Parameters	Value
Repetition time (TR)	1500 ms
Echo time (TE)	35 ms
Number of excitations (NEX)	1
Phase	18
Frequency	18
Volumes of interest (VOIs)	10 × 8 × 2 cm^3^

**Table 3:** The neuropsychiatric manifestations occurring in NPSLE patients.

Neuropsychiatric manifestations	Number
Myelitis	1
Seizure disorder	9
Severe headache	5
Stroke	3
Peripheral polyneuropathy	2
Acute confusional state	1
Anxiety	2

**Table 4:** The demographic characters of the NPSLE patients and healthy controls.

	Number	Mean age (year)	Sex (male/female)
NPSLE	23	31.71	2/14
HC	16	29.5	5/18
*p* value	—	0.765^∗^	0.018^∗^

^∗^Student's *t*-test.

**Table 5:** Changes of major metabolite features detected in ^1^H-MRS in NPSLE patients vs. healthy controls.

Brain region	Metabolites	HCs (*n* = 16)	NPSLE (*n* = 23)	*p* value	AUC
RPCG	Cr	2.304 ± 0.473	3.124 ± 0.418	0.274	0.591
	PCr^∗^	4.184 ± 0.651	2.479 ± 0.583	0.035	0.588
	Cr+PCr	3.465 ± 0.506	2.900 ± 0.491	0.391	0.432
	NAA	4.457 ± 0.428	3.935 ± 0.419	0.478	0.457
	NAAG^∗∗^	3.604 ± 0.550	3.782 ± 0.581	0.848	0.548
	NAA+NAAG	6.222 ± 0.728	4.996 ± 0.692	0.183	0.283
	NAA+NAAG/Cr+PCr	3.622 ± 0.776	5.316 ± 0.649	0.086	0.62
	mI	2.075 ± 0.388	2.535 ± 0.335	0.274	0.557
	mI/Cr+PCr^∗∗^	2.626 ± 0.473	3.437 ± 0.399	0.165	0.567
	Cho+PCh^∗^	5.151 ± 0.477	3.892 ± 0.502	0.039	0.492
	Cho+PCh/Cr+PCr^∗∗^	4.138 ± 0.535	4.867 ± 0.377	0.249	0.466
	Glu+Gln	4.799 ± 0.503	4.452 ± 0.407	0.322	0.398
	Glu+Gln/Cr+PCr	4.296 ± 0.494	4.253 ± 0.456	0.953	0.389

LPCG	Cr	4.211 ± 0.591	3.452 ± 0.491	0.442	0.485
	PCr	4.248 ± 0.758	4.125 ± 0.602	0.825	0.435
	Cr+PCr^∗∗^	2.803 ± 0.620	3.788 ± 0.506	0.225	0.41
	NAA	4.208 ± 0.340	3.395 ± 0.319	0.104	0.401
	NAAG	4.681 ± 0.321	3.817 ± 0.437	0.249	0.444
	NAA+NAAG^∗∗∗^	7.005 ± 0.497	6.009 ± 0.333	0.005	0.66
	NAA+NAAG/Cr+PCr^∗∗∗^	7.655 ± 0.199	6.571 ± 0.259	0.006	0.658
	mI^∗^	7.284 ± 0.207	6.273 ± 0.293	0.009	0.62
	mI/Cr+PCr^∗∗^	6.893 ± 0.167	6.513 ± 0.159	0.132	0.413
	Cho+PCh	6.303 ± 0.354	6.395 ± 0.369	0.636	0.391
	Cho+PCh/Cr+PCr	8.776 ± 0.201	7.673 ± 0.486	0.076	0.457
	Glu+Gln^∗∗^	5.625 ± 0.898	6.886 ± 0.513	0.595	0.682
	Glu+Gln/Cr+PCr	5.601 ± 0.174	5.385 ± 0.264	0.745	0.457

RDT	Cr	5.898 ± 0.163	5.931 ± 0.226	0.859	0.535
	PCr^∗^	9.658 ± 0.691	8.485 ± 0.431	0.008	0.694
	Cr+PCr^∗^	10.614 ± 0.269	9.178 ± 0.316	0.001	0.747
	NAA^∗^	8.094 ± 0.265	6.612 ± 0.314	0.002	0.76
	NAAG	8.705 ± 0.266	7.739 ± 0.382	0.058	0.576
	NAA+NAAG	7.660 ± 0.333	7.239 ± 0.427	0.636	0.435
	NAA+NAAG/Cr+PCr^∗^	9.645 ± 0.271	7.703 ± 0.529	0.004	0.639
	mI	7.552 ± 1.146	8.459 ± 0.549	0.496	0.66
	mI/Cr+PCr^∗^	8.821 ± 0.195	7.907 ± 0.331	0.041	0.766
	Cho+PCh^∗^	9.435 ± 0.245	7.537 ± 0.374	0.001	0.776
	Cho+PCh/Cr+PCr	0.753 ± 0.173	0.852 ± 0.207	0.79	0.5
	Glu+Gln	0.830 ± 0.268	0.896 ± 0.219	0.767	0.478
	Glu+Gln/Cr+PCr	0.501 ± 0.197	0.618 ± 0.217	0.767	0.457

LDT	Cr	2.163 ± 0.388	1.821 ± 0.378	0.221	0.591
	PCr	0.429 ± 0.263	0.776 ± 0.247	0.188	0.435
	Cr+Cr	1.005 ± 0.343	0.744 ± 0.326	0.274	0.457
	NAA^∗∗^	0.923 ± 0.344	1.594 ± 0.275	0.076	0.639
	NAAG^∗∗∗^	1.242 ± 0.161	0.559 ± 0.118	0.002	0.673
	NAA+NAAG	1.272 ± 0.214	1.010 ± 0.223	0.255	0.598
	NAA+NAAG/Cr+PCr^∗^	10.263 ± 0.725	9.027 ± 0.449	0.003	0.735
	mI^∗^	11.241 ± 0.256	9.843 ± 0.341	0.002	0.60
	mI/Cr+PCr^∗^	8.411 ± 0.204	6.993 ± 0.349	0.002	0.779
	Cho+PCh^∗∗^	10.063 ± 0.258	8.924 ± 0.224	0.002	0.685
	Cho+PCh/Cr+PCr	7.884 ± 0.328	7.748 ± 0.465	0.929	0.435
	Glu+Gln^∗^	10.173 ± 0.233	8.251 ± 0.492	0.001	0.548
	Glu+Gln/Cr+PCr	8.047 ± 1.213	9.348 ± 0.544	0.615	0.629

RLN	Cr^∗^	9.868 ± 0.231	8364 ± 0.362	0.005	0.648
	PCr^∗∗∗^	10.409 ± 0.309	8.383 ± 0.389	0.001	0.673
	Cr+PCr^∗∗^	1.394 ± 0.098	1.555 ± 0.068	0.478	0.5
	NAA	1.501 ± 0.055	1.556 ± 0.068	0.359	0.5
	NAAG	1.163 ± 0.024	1.112 ± 0.029	0.515	0.5
	NAA+NAAG	1.508 ± 0.047	1.410 ± 0.047	0.225	0.5
	NAA+NAAG/Cr+PCr	1.323 ± 0.066	1.215 ± 0.079	0.478	0.5
	mI^∗^	1.170 ± 0.029	1.044 ± 0.058	0.036	0.5
	mI/Cr+PCr	1.118 ± 0.172	1.358 ± 0.077	0.701	0.603
	Cho+PCh^∗∗∗^	1.801 ± 0.032	1.611 ± 0.048	0.003	0.588
	Cho+PCh/Cr+PCr^∗∗∗^	1.800 ± 0.067	1.461 ± 0.067	0.003	0.598
	Glu+Gln	5.246 ± 0.447	4.649 ± 0.582	0.231	0.482
	Glu+Gln/Cr+PCr^∗∗^	5.429 ± 0.512	5.604 ± 0.313	0.836	0.432

LLN	Cr	4.839 ± 0.442	5.584 ± 0.395	0.107	0.457
	PCr^∗^	4.220 ± 0.296	5.574 ± 0.390	0.008	0.641
	Cr+PCr	3.167 ± 0.687	4.850 ± 0.692	0.076	0.501
	NAA	5.630 ± 0.443	5.205 ± 0.608	0.953	0.504
	NAAG	3.816 ± 0.642	5.529 ± 0.545	0.124	0.526
	NAA+NAAG	5.370 ± 0.249	5.819 ± 0.503	0.722	0.557
	NAA+NAAG/Cr+PCr	5.729 ± 0.304	5.901 ± 0.366	0.88	0.651
	mI^∗^	0.703 ± 0.055	5.674 ± 0.071	0.894	0.5
	mI/Cr+Cr^∗∗^	0.708 ± 0.628	5.838 ± 0.034	0.036	0.478
	Cho+PCh^∗∗∗^	0.655 ± 0.061	0.883 ± 0.061	0.007	0.601
	Cho+PCh/Cr+PCr^∗∗∗^	0.610 ± 0.038	0.844 ± 0.055	0.001	0.541
	Glu+Gln	0.480 ± 0.094	1.033 ± 0.395	0.132	0.435
	Glu+Gln/Cr+PCr	0.611 ± 0.029	0.667 ± 0.085	0.344	0.5

RI	Cr^∗^	0.532 ± 0.095	0.786 ± 0.062	0.005	0.603
	PCr	0.944 ± 0.037	1.109 ± 0.067	0.193	0.5
	Cr+PCr	0.982 ± 0.047	1.016 ± 0.044	0.478	0.5
	NAA^∗^	1.322 ± 0.093	1.179 ± 0.072	0.023	0.457
	NAAG	1.667 ± 0.050	1.548 ± 0.078	0.124	0.5
	NAA+NAAG	2.107 ± 0.071	2.051 ± 0.019	0.615	0.5
	NAA+NAAG/Cr+PCr^∗∗^	1.983 ± 0.061	1.893 ± 0.063	0.261	0.5
	mI	1.561 ± 0.094	1.668 ± 0.012	0.209	0.466
	mI/Cr+PCr	2.285 ± 0.696	2.153 ± 0.131	0.626	0.5
	Cho+PCh^∗∗^	1.496 ± 0.240	1.872 ± 0.138	0.329	0.603
	Cho+PCh/Cr+PCr	2.056 ± 0.088	1.895 ± 0.098	0.425	0.5
	Glu+Gln	2.127 ± 0.068	2.034 ± 0.076	0.461	0.5
	Glu+Gln/Cr+PCr	0.176 ± 0.012	0.201 ± 0.011	0.359	0.5

RPWM	Cr	0.220 ± 0.008	0.232 ± 0.008	0.442	0.5
	PCr^∗^	0.292 ± 0.007	0.330 ± 0.011	0.008	0.5
	Cr+PCr	0.292 ± 0.006	0.292 ± 0.005	0.859	0.5
	NAA	0.253 ± 0.011	0.261 ± 0.018	0.657	0.5
	NAAG	0.260 ± 0.006	0.274 ± 0.017	0.274	0.5
	NAA+NAAG	0.201 ± 0.030	0.263 ± 0.013	0.174	0.5
	NAA+NAAG/Cr+PCr	0.381 ± 0.015	0.372 ± 0.018	0.723	0.5
	mI	0.369 ± 0.012	0.355 ± 0.013	0.329	0.5
	mI/Cr+PCr	15.876 ± 1.396	14.623 ± 0.850	0.165	0.622
	Cho+PCh	15.208 ± 1.396	14.357 ± 0.687	0.132	0.495
	Cho+PCh/Cr+PCr^∗∗∗^	15.249 ± 0.636	11.941 ± 1.002	0.01	0.576
	Glu+Gln	11.651 ± 0.901	13.566 ± 0.755	0.261	0.432
	Glu+Gln/Cr+PCr^∗∗^	8.923 ± 1.758	12.151 ± 1.041	0.104	0.535

LPWM	Cr^∗^	17.459 ± 0.726	14.326 ± 1.757	0.005	0.726
	PCr^∗∗^	10.796 ± 1.628	14.001 ± 0.975	0.375	0.735
	Cr+PCr	11.579 ± 0.701	10.167 ± 0.996	0.79	0.704
	NAA	11.621 ± 0.585	11.756 ± 0.604	0.813	0.457
	NAAG^∗^	2.113 ± 0.163	2.569 ± 0.145	0.036	0.5
	NAA+NAAG^∗∗^	2.028 ± 0.149	2.267 ± 0.138	1	0.5
	NAA+NAAG/Cr+PCr^∗∗^	2.089 ± 0.083	1.855 ± 0.154	0.225	0.5
	mI^∗^	1.668 ± 0.119	2.094 ± 0.116	0.033	0.516
	mI/Cr+PCr	1.526 ± 0.288	1.788 ± 0.147	0.679	0.548
	Cho+PCh	2.007 ± 0.097	1.875 ± 0.244	0.132	0.603
	Cho+PCh/Cr+PCr	1.499 ± 0.236	1.996 ± 0.117	0.091	0.603
	Glu+Gln	2.004 ± 0.077	1.925 ± 0.159	0.515	0.5
	Glu+Gln/Cr+PCr	2.028 ± 0.079	2.153 ± 0.158	0.813	0.5

^∗^Metabolites with significant difference (*p* < 0.05). ^∗∗^Metabolites for our model. ^∗∗∗^Metabolites for our model and metabolites with significant difference (*p* < 0.05).

## Data Availability

The ^1^H-MRS data used to support the findings of this study have not been made available for the private protection of patient information.
